# Single Crystal Growth and Spin Polarization Measurements of Diluted Magnetic Semiconductor (BaK)(ZnMn)_2_As_2_

**DOI:** 10.1038/s41598-017-08394-z

**Published:** 2017-11-03

**Authors:** G. Q. Zhao, C. J. Lin, Z. Deng, G. X. Gu, S. Yu, X. C. Wang, Z. Z. Gong, Yasutomo J. Uemura, Y. Q. Li, C. Q. Jin

**Affiliations:** 1 Institute of Physics, Chinese Academy of Sciences; Collaborative Innovation Center of Quantum Matter, Beijing, 100190 China; 2 University of Chinese Academy of Sciences, Beijing, 100190 China; 3 Department of Physics, Columbia University, New York, NY 10027 USA

**Keywords:** Electronic devices, Magnetic properties and materials

## Abstract

Recently a new diluted magnetic semiconductor, (Ba,K)(Zn,Mn)_2_As_2_ (BZA), with high Curie temperature was discovered, showing an independent spin and charge-doping mechanism. This makes BZA a promising material for spintronics devices. We report the successful growth of a BZA single crystal for the first time in this study. An Andreev reflection junction, which can be used to evaluate spin polarization, was fabricated based on the BZA single crystal. A 66% spin polarization of the BZA single crystal was obtained by Andreev reflection spectroscopy analysis.

## Introduction

Diluted magnetic semiconductors (DMSs) have attracted research attention because of their physical properties and applications for spintronics devices since the discovery of (Ga,Mn)As film by H. Ohno in the 1990s^1–7^. In these III–V DMSs, such as (Ga,Mn)As and (In,Mn)As, divalent Mn substitution into trivalent Ga (or In) sites leads to severely limited chemical solubility, resulting in metastable specimens that only exist as epitaxial thin films^2^. The heterovalent substitution, which simultaneously dopes hole carriers and spins, makes the flexible tuning of quantum freedom i.e., the individual control of charge and spin concentrations, difficult in DMS. To solve these problems, several new types of DMSs with independent spin or charge doping were synthesized. Examples include “111” type Li(Zn,Mn)As, “122” type (Ba,K)(Zn,Mn)_2_As_2_ (BZA), and “1111” type (La,Ca)(Zn,Mn)SbO, which are named by the chemical ratio of their parent phases^8–28^.

Among the new DMSs, the ThCr_2_Si_2_-type BZA has a Curie temperature (*T*c) of up to 230 K, which marks the current reliable record *T*c for DMSs where ferromagnetism is mediated by carriers^12,13^. BZA is one of the milestones in DMS research^28^. A robust nearest-neighbor ferromagnetic correlation that exists above the ferromagnetic ordering temperature suggested the potentialof discovering a higher *T*c in further study^25^. Angle-resolved photoemission spectroscopy showed a clear impurity band of Mn-doping well below the Fermi energy^26,27^. Besides, the excellent match of lattice parameters (within 5% mismatch) among “122” type DMS BZA, “122” iron-based superconductor (Ba,K)Fe_2_As_2_, and antiferromagnetic BaMn_2_As_2_ is promising for fabricating heterojunctions with different types of ordering^13^. Thus, BZA provides a unique opportunity to elucidate the intrinsic physics of DMSs, and their physically transparent description may also be general and applicable to other DMS materials^24,28,29^. For both fundamental understanding and potential applications on spintronicss devices, direct measurement of spin polarization (*P*) in BZA is an important parameter. The Andreev reflection (AR) technique has been applied to measure the spin polarization rate of prototypical III-V based DMS, such as 85% for (Ga,Mn)As^30^, 57% ± 5% for (Ga,Mn)Sb^31^ and 72% for (In,Mn)As^32^. Single crystals with various K- and Mn-doping levels have been grown, and the *T*c of crystals are controlled with K and Mn concentrations, that is, carrier and spin density, respectively. As an initial attempt, selecting a (Ba_0.904_K_0.096_)(Zn_0.805_Mn_0.195_)_2_As_2_ crystal that shows good shape and size allows the fabrication of Andreev reflection junction. In this study, we report the basic properties of (Ba_0.904_K_0.096_)(Zn_0.805_Mn_0.195_)_2_As_2_ single crystal and the degree of spin polarization obtained from the crystal-based Andreev reflection spectroscopy.

## Results and Discussion

### Chemical composition and crystal structure

**Figure 1:**
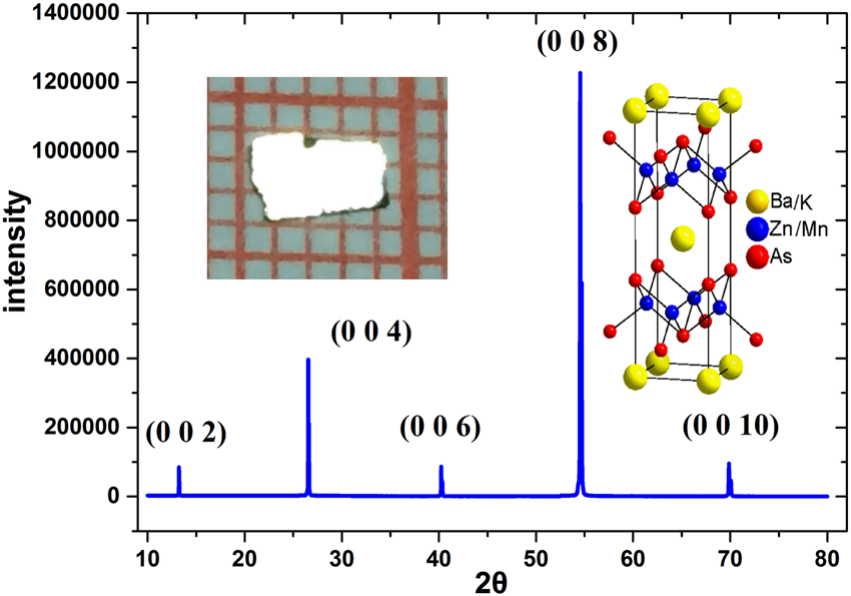
The X-ray diffraction patterns of (Ba_0.904_K_0.096_)(Zn_0.805_Mn_0.195_)_2_As_2_ were collected at room temperature. The inset shows the crystal structure (right) and its photograph (left).

Chemical compositions and morphology of the single crystal were investigated through energy dispersive X-ray analysis (EDX) and inductively coupled plasma (ICP) mass spectrometry. The real atom ratio, (Ba_0.904_K_0.096_)(Zn_0.805_Mn_0.195_)_2_As_2_, was determined by ICP. We also used EDX to analyze the real atom ratio and the doping homogeneity, and its results were consistent with the ICP results. Figure 1 shows the obtained BZA crystals with a typical size of 3 × 3 mm^2^. The X-ray diffraction patterns of the obtained crystals only show the (002n) peaks of the BZA structure as illustrated in Fig. 1. The unit cell constants are calculated as c = 13.4658(6) Å, which is consistent with previous reports^13^. To further confirm the phase, single crystals were ground to conduct powder X-ray diffraction. The obtained pattern fits well with the structure of ThCr_2_Si_2_.

### Magnetic properties

**Figure 2:**
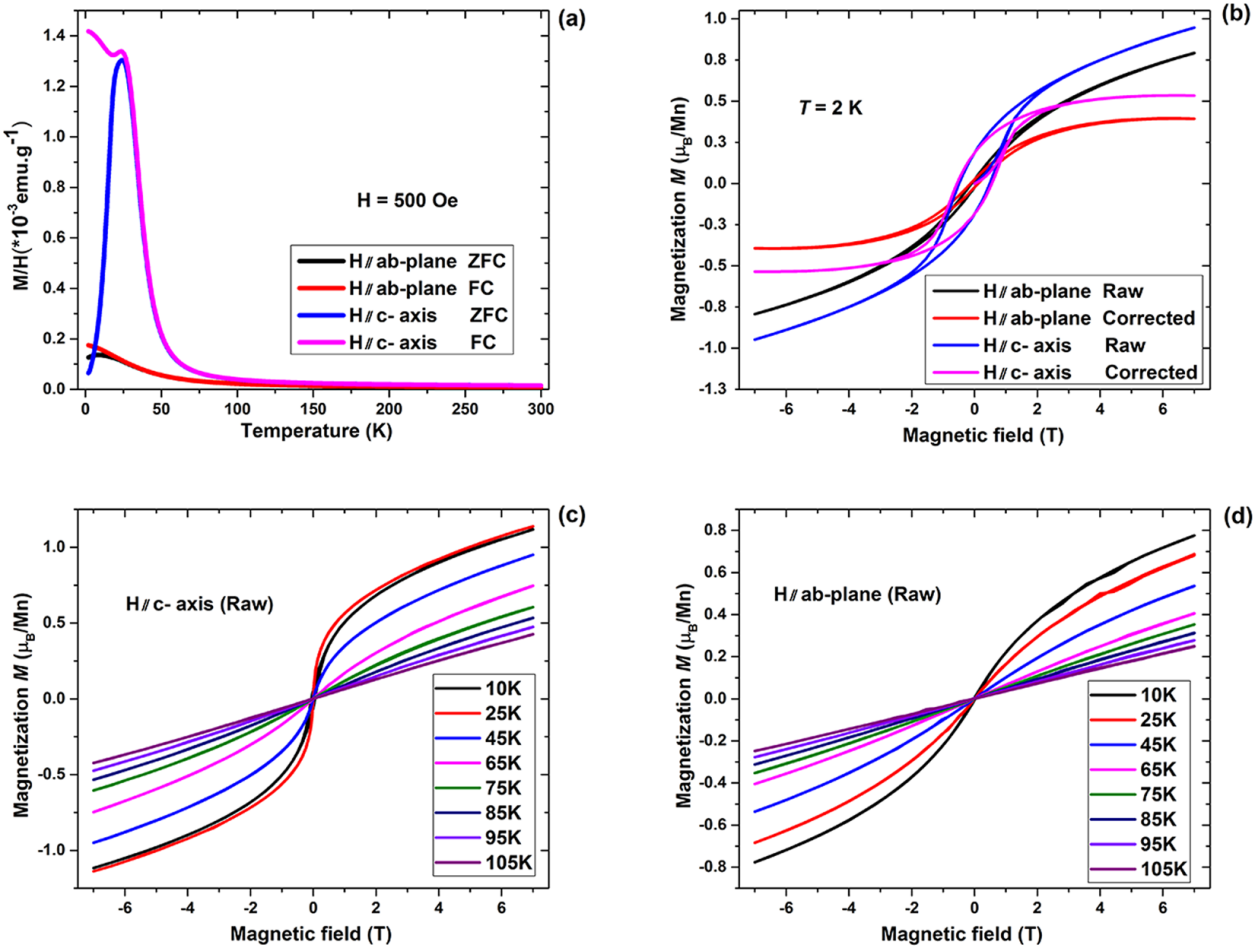
Magnetic properties of (Ba_0.904_K_0.096_)(Zn_0.805_Mn_0.195_)_2_As_2_ and its anisotropy. (**a**) DC magnetization measured along c-axis and ab-plane with ZFC and FC mode under external field *H* = 500 Oe. (**b**) The hysteresis curves M(H) measured at 2 K in deferent axis to exhibit magnetic anisotropy. (**c**) and (**d**) The hysteresis curves M(H) measured at selected temperatures from 10 K to 105 K in c-axis and ab-plane.

The DC magnetic susceptibility of the BZA single crystal was characterized using a superconducting quantum interference device magnetometer (Quantum Design) in both zero-field-cooling (ZFC) and field-cooling (FC) modes. Both in-plane magnetization *M* versus *T* data (M_ab_(T)) and the *H* // *c* axis M_c_(T) at *H = *500 Oe, shown in Fig. 2(a), exhibit clear ferromagnetic enhancements at around 50 K. A precise determination of *T*c can be done via critical exponent analysis, which requires a fine measurement of M-H data in a sufficiently small temperature interval over a large temperature region. Ferromagnetism is also evident from the *M*(*H*) plots shown in Fig. 2(b) inset with a saturation moment *M*_*sat*_ of about 0.5 and 0.3 (±0.03) μ_B_/Mn in M_c_(H) and M_ab_(H), respectively. The *M*_*sat*_ is defined as high-field *M*(*H*) data at 2 K after subtracting the small T-linear component^6^. As discussed in our previous paper on polycrystalline samples, the antiferromagnetic coupling of Mn to the nearest neighboring Zn sites can reduce the saturation moment and also cause a linear component on the M(H) curves simultaneously^18^. The small T-linear component of a current single crystal is calculated at 0.059 and 0.057 μ_B_/T along the c-axis and the ab-plane, respectively. The coercive forces, *H*_*c*_^*c*^ in M_c_(H) and *H*_*c*_^*ab*^ in M_ab_(H), are about 5300 and 1200 Oe. The values of *H*_*c*_^*c*^ and the *H*_*c*_^*ab*^ become smaller when temperature rises while *M*_*sat*_ values along the c-axis are always larger than those in the ab-plane at any temperature from 10 to 105 K, as shown in Fig. 2(c) and (d), respectively. The crystals show clear anisotropic behavior with easy axis along c from the measurements of M(T) and M(H).

### Electrical transport properties

**Figure 3:**
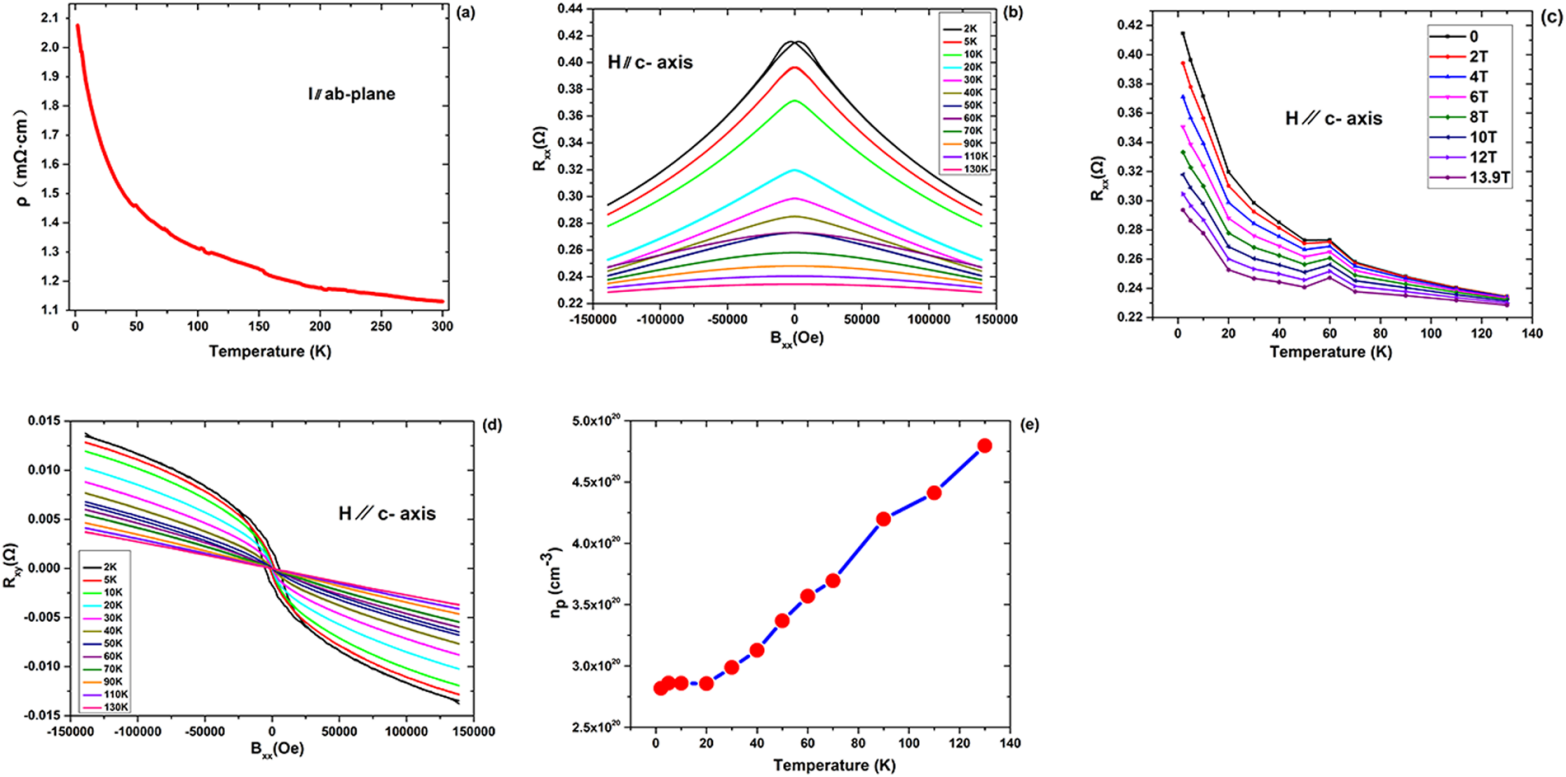
Transport properties of (Ba_0.904_K_0.096_)(Zn_0.805_Mn_0.195_)_2_As_2_ single crystal. (**a**) The temperature dependence of resistivity with current in ab-plane. (**b**) The magnetoresistance R_xx_ at several selected temperatures from 2 K to 130 K are presented. (**c**) The temperature dependence of the MR were plotted in various field strengths. (**d**) The anomalous Hall effect R_xy_ at several selected temperatures from 2 K to 130 K are presented. (**e**) The temperature dependence of the carrier density calculated based on R_xx_ and R_xy_ are shown.

Figure 3(a) shows the temperature dependence of resistivity with electrical current in the ab-plane (ρ_ab_(T)). Resistivity grows as temperature falls by virtue of the semiconductor behavior and localization effect^33^. Magnetoresistance (MR) and Hall effect measurements were performed with the electrical current in the ab-plane ranging from 2 to 130 K and with the magnetic field parallel to the c-axis of up to 14 T. Figure 3(b) shows the change of (MR-R_xx_) at several selected temperatures from 2 to 130 K, and Fig. 3(d) shows the corresponding Hall resistance, R_xy_. The negative slope in Hall resistance at high magnetic field indicates a p-type carrier, which is consistent with the substitution of monovalent K into divalent Ba. The salient features of R_xx_ and R_xy_ are the gradual emergence of hysteresis at temperatures below 10 K, from which a coercive field *H*_*c*_^*c*^ around 5300 Oe can be clearly identified at 2 K, which agrees well with the magnetization measurement shown in Fig. 2(b). Based on the transport measurements, we observed non-linear Hall resistance at low magnetic field of up to 70 K. The Hall resistance above 70 K becomes linear, which suggests spin correlation effect does not occur. However, this temperature was not necessarily similar to the ferromagnetic transition (long-range order) temperature if a region with short-range spin correlation exists, such as in GaMnAs^1^ and in BZA^25^. Therefore, 50 and 70 K represent two emergence points with different types of spin correlation for long-range ordering and short-range fluctuations, respectively. In addition, we also noticed an MR-R_xx_ “overlap” between 50 and 60 K, which is near *T*c as shown in Fig. 3(b). The “overlap” results from the sudden reduction of MR above *T*c of 60 K, which is easily identifiable in Fig. 3(c). This phenomenon was also observed in (Ga,Mn)As^1,34^.

To determine the carrier density for BZA, we made a more quantitative analysis of hall resistance. Generally, the scattering from the magnetic ions in the ferromagnetic statue of a DMS material causes the carriers to accumulate asymmetrically in the transverse direction relative to the electric current, giving an additional contribution to the normal Hall effect, which is called anomalous Hall effect^33^. The Hall resistance, therefore, can be phenomenologically expressed as

1 $${\rm{R}}{\rm{x}}{\rm{y}}{={\rm{R}}}_{{\rm{0}}}{{\rm{B}}+{\rm{R}}}_{{\rm{s}}}{\rm{M}}({\rm{B}}),$$

where R_0_ is the ordinary Hall coefficient; R_s_ is the anomalous Hall coefficient; and M is the magnetization moment. As mentioned, a small paramagnetic background occurs during field-dependent magnetization measurements at low temperatures in this material, and the magnetization saturates only until the magnetic field reaches ~11–14 T. In Fig. 3(c), the R_xy_ at these high-field regions are almost straight lines, implying the dominance of a single-type carrier near the Fermi surface responding to the magnetic field. Therefore, a single band model of Eq. 1 justifies the Hall effect analysis in BZA. As the magnetization saturates at a high magnetic field, the anomalous Hall resistance R_s_M(B) becomes independent of the magnetic field, and we can deduce the ordinary Hall coefficient R_0_, which is simply equivalent to the high-field slope of R_xy_ in Fig. 3(c). Then, the hole carrier density n_p_ for every temperature can be deduced from the relation n_p_ = 1/e|R_0_|. Figure 3(d) shows the plot of n_p_ versus T, where the carrier density increases monotonically from 2.82 × 10^20^ cm^−3^ at 2 K to 4.80 × 10^20^ cm^−3^ at 130 K. Highly similar to a semiconducting behavior, the observed increase of carrier density under elevated temperatures may arise from the enhanced thermal excitation of carriers from the impurity band to the conduction band.

### Spin polarization

**Figure 4:**
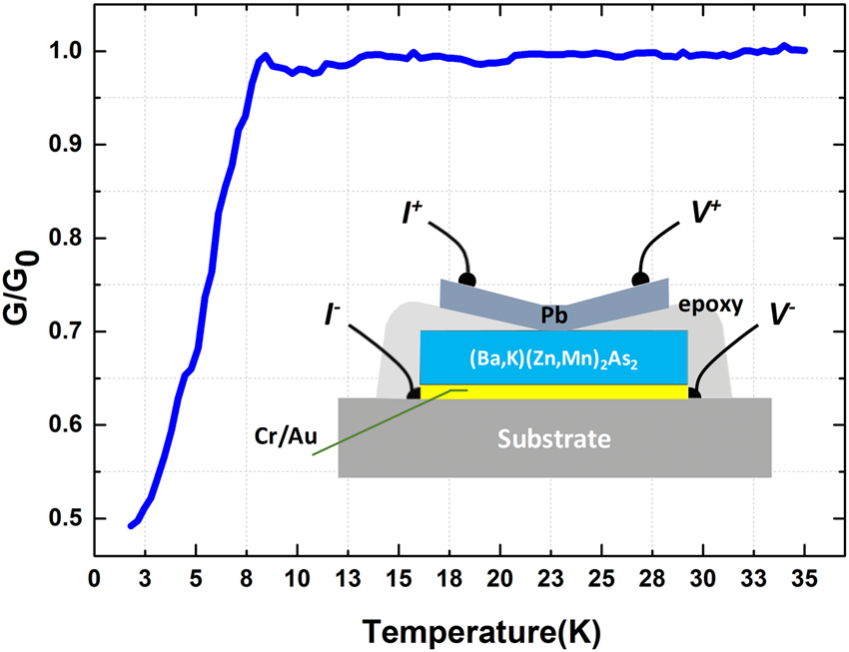
Sketch of the (Ba_0.904_K_0.096_)(Zn_0.805_Mn_0.195_)_2_As_2_/Pb junctions used for Andreev reflection spectroscopy. The inset is the normalization for the differential conductance G/G_0_.

**Figure 5:**
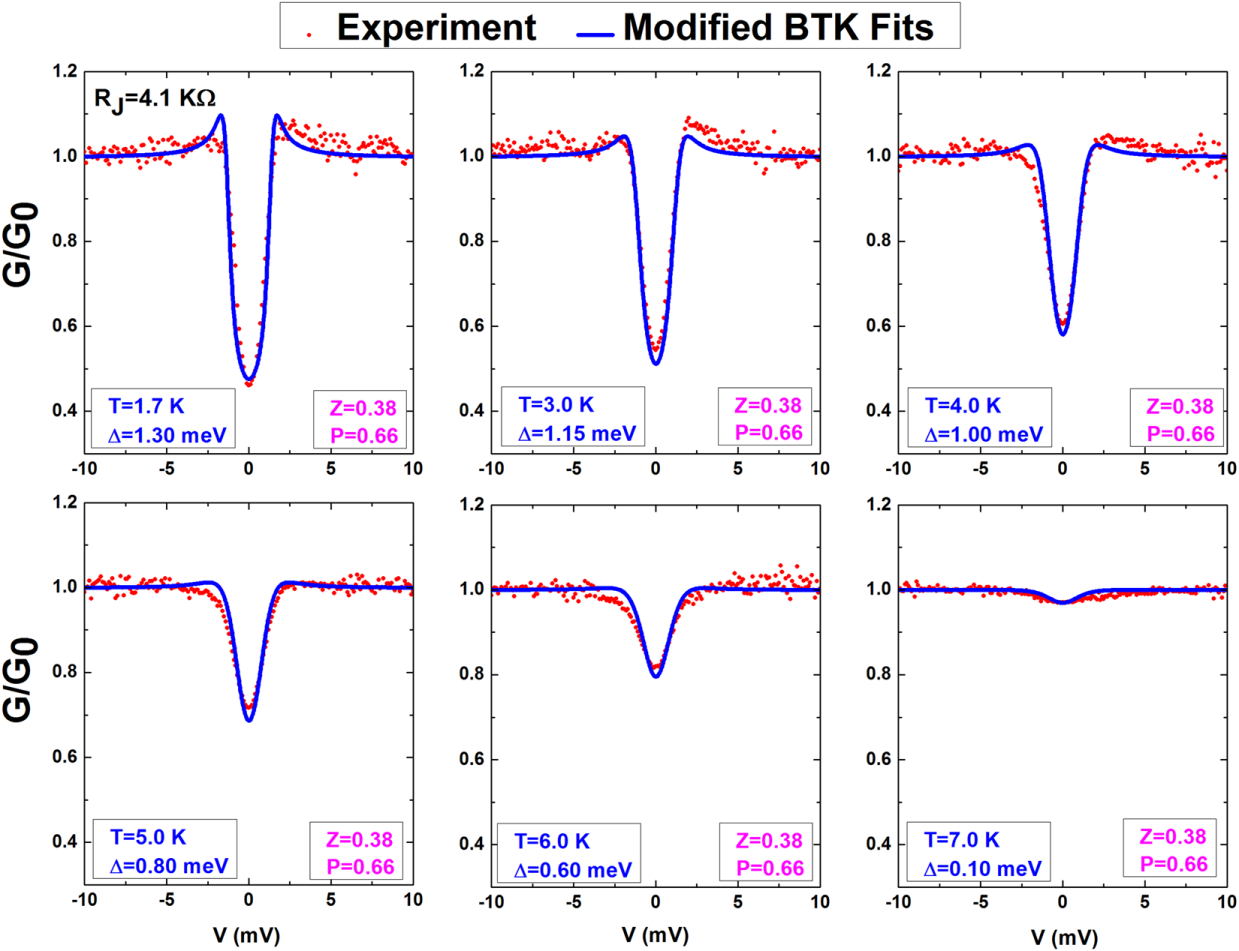
Normalized differential conductance G/G_0_ spectra (red dot) and their fits to the modified BTK theory (blue line) at selected temperatures from 1.7 K to 7.0 K. The standard error for the fitting data been evaluated is around 3.6% for *T* = 1.7 K and decrease to 0.7% for *T* = 7 K.

The spin polarization rate is one of the key parameters of DMS for direct fundamental and applied relevance. *P* values of various traditional DMS materials have been determined by analysis of Andreev reflection spectroscopy. Similarly, we use Andreev reflection spectroscopy to directly probe the electron spin polarization in the BZA single crystal. This method is successfully applicable in measuring the spin polarization in (Ga,Mn)As^30^, (Ga,Mn)Sb^31^, and (In,Mn)As^32^ along with other ferromagnetic materials, such as (La,Sr)MnO_3_^35^, CrO_2_^36^, EuS^37^, and HgCr_2_Se_4_^38^. The inset of Fig. 4 shows a schematic view of the BZA/Pb junction. The typical junction area is around 100 × 100 μm^2^. The differential conductance, defined as G(*V*) = dI(*V*)/d*V*, was measured as a function of dc-bias voltage (*V*) crossing the junction by using phase-sensitive lock-in techniques. The amplitude of the ac modulation output from the lock-in amplifier was kept around 20 nA, which is sufficiently small to avoid spurious artificial effects. Normalization of the differential conductance G to G_0_ was conducted with G_0_ at a magnetic field of 0.25 T. In Fig. 4, we present the temperature dependence of G/G_0_ from 1.7 to 35 K, where a dramatic drop appears at T = 7.2 K. This temperature corresponds exactly to the superconducting phase transition of Pb, and the dropping of G/G_0_ confirms that the Andreev reflection process occurs at the interface between BZA single crystal and the superconducting Pb film. From the plot of G/G_0_ versus dc-bias *V* in Fig. 5 with several temperatures from 1.7 to 7 K, we also observed a suppression of the Andreev reflection spectra inside the superconducting gap, which can be attributed to the ferromagnetism originating from spin-imbalanced density of states around the Fermi level in BZA. The imbalance of spin density partially inhibits the formation of Cooper pairs and their tunneling into the superconductor.

We used the modified Blonder–Tinkham–Klapwijk (BTK) theory^39^ to quantitatively describe the electron tunneling process at interface between ferromagnetic material and s-wave superconductor. Taking account two basic processes: Andreev reflection and normal reflection, current density *I*_tot_ across the interface can be described by Eq. 2,

2 $${I}_{{\rm{tot}}}=(1-|P|){I}_{{\rm{u}}}+|P|{I}_{{\rm{p}}}$$

where the spin unpolarized current *I*_u_ and spin polarized current *I*_p_ are separately taken into account with a weight of the spin polarization *P*, which is defined as *P* = (N↑ − N↓)/(N↑ + N↓) where N↑/N↓ is the density of state for spin up/down band. For each current *I*_u_ and *I*_p_, it takes

3 $$I=2{\rm{e}}AN{{\rm{\nu }}}_{F}{\int }^{}(f(E-V))-f(E)(1+{A}_{u}(E)-{B}_{u}(E)){\rm{d}}E$$

4 $$I=2{\rm{e}}AN{{\rm{\nu }}}_{F}{\int }^{}(f(E-V))-f(E)(1+{A}_{p}(E)-{B}_{p}(E)){\rm{d}}E$$

where *e* is the electron charge; *A* is the junction area; *N* is the density of state around Fermi level *E*_F_; *ν*_F_ is the Fermi velocity; *f*(*E*) is the Fermi-Dirac distribution at temperature of *T*; *V* is the biase voltage across the junction; *A*_*u*_ and *B*_*u*_ are respectively the Andreev reflection and normal reflection probability for spin unpolarized current while *A*_*p*_ and *B*_*p*_ are corresponding polarized current respectively. Table 1 lists the expressions of *A*_*p*_, *B*_*p*_, *A*_*u*_, and *B*_*u*_. The deviative of *I*_tot_ with biase voltage *V* to attain the conductivity *G*(*V*) (*G*(*V*) = d*I*_tot_/d*V*) with a final normalization to *G*_0_ from |*E|* >> Δ limit, results *G*(*V*)/G_0_:

5 $$\frac{G(V)}{{G}_{0}}=\frac{1}{{G}_{0}}\frac{d{I}_{tot}}{dV}=\frac{1}{{G}_{0}}[(1-|P|)\frac{d{I}_{u}}{dV}+|P|\frac{d{I}_{p}}{dV}],\,{G}_{0}\equiv \mathop{\mathrm{lim}}\limits_{|E| >  > \Delta }\frac{d{I}_{tot}}{dV}$$

**Table 1:** Probabilities of Andreev and normal reflection of polarized (A_*p*_ and B_*p*_) and unpolarized (A_*u*_ and B_*u*_) currents in modified BTK model. Δ is the superconducting gap of Lead; *Z* is interface barrier height; ${\rm{\gamma }}={u}_{0}^{2}+{({u}_{0}^{2}-{{\rm{\nu }}}_{0}^{2})}^{2}{Z}^{2}$ and ${u}_{0}^{2}=1-{{\rm{\nu }}}_{0}^{2}=\,[1+\sqrt{({E}^{2}-{\Delta }^{2})/{E}^{2}}]/2$, where ${u}_{0}$ and ${\nu }_{0}$ are BCS coherence factors evaluated on the branch outside of the Fermi surface^39^.

	Unpolarized currents		Polarized currents	
	A_*u*_	B_*u*_	A_*p*_	B_*p*_
|*E*| = *|*e*V*| < Δ	$$\frac{\,{\Delta }^{2}}{{E}^{2}-({\Delta }^{2}-{E}^{2}){(1+2{Z}^{2})}^{2}}$$	$$1-{{\rm{A}}}_{u}$$	0	1
|*E*| = |e*V*| > Δ	$$\frac{{u}_{0}^{2}{v}_{0}^{2}}{{\gamma }^{2}}$$	$$\frac{{({u}_{0}^{2}-{v}_{0}^{2})}^{2}{Z}^{2}(1+{Z}^{2})}{{\gamma }^{2}}$$	0	$$\frac{{({u}_{0}^{2}-{v}_{0}^{2})}^{2}{Z}^{2}(1+{Z}^{2})}{{\gamma }^{2}}$$

Figure 5 presents the fitting curves along with the experimental data for comparison with all data that fit well in the entire temperature range and the extracted fitting parameters. We only found the superconducting gap, Δ, as a decreasing behavior in elevated temperatures from the fitting result with different temperatures, strongly suggesting the determination of parameters, *P* and *Z*, in the fitting process and the temperature dependence of Δ, *Z*, and *P* are all behaved as expected behavior from the BTK theory. The curve fittings have also resulted in a small *Z* value (Z = 0.38 << 1), which implies the manifestation of a clean and transparent interface between BZA crystal and Pb film in our junctions. A small *Z* value is a crucial requirement in spectral analysis because it directly warrants the reliability and accuracy for extracting the spin polarization in the fitting process, our result on *Z* further confirms the determination of spin polarization *P* in our Andreev reflection spectra analysis, which is 66% ± 1% for the BZA single crystal.

## Conclusions

We have successfully grown the (Ba,K)(Zn,Mn)_2_As_2_ single crystal for the first time. The crystal shows a ferromagnetic transition with easy magnetization axis along the c-axis. The carrier density is determined from the anomalous Hall effect from 2.82 × 10^20^ to 4.80 × 10^20^ cm^−3^ as the temperature increases from 2 to 130 K. More significantly, the Andreev reflection junction from the selected large single crystal was fabricated to testify spin polarization degree of BZA, and 66% spin polarization was reached. The success on Andreev reflection junction paves a solid route for fabricating multilayer junctions based on BZA DMS.

## Method

Single (Ba_0.904_K_0.096_)(Zn_0.805_Mn_0.195_)_2_As_2_ crystal was grown via the flux technique. Precursor materials of (Zn,Mn)As mixture were first prepared with high-purity Zn, Mn, and As in a sealed tube. The samples were heated at 750 °C and held for several hours before cooling down to room temperature. Mixtures of precursors with high-purity Ba and K in appropriate molar ratio were loaded into the niobium tube with argon under 1 atm pressure before sealing into a quartz tube. The process was handled in a glove box with high-purity argon to protect the materials from reacting with air or water. The quartz tube was heated at 1200 °C and held for several hours before cooling down to room temperature at a rate of 3 °C/h. The recovered samples were characterized by X-ray powder diffraction with a Philips X’pert diffractometer using Cu-*Kα* radiation. Real compositions were determined by using EDX on a commercial scanning electron microscope and ICP mass spectrometry. The DC magnetic properties were examined by using a Superconductivity Quantum Interference Device (Quantum design), and transport properties and Andreev reflection junction were observed by a Physical Property Measurement System (Quantum design) with lock-in techniques. During the transport experiments, the single crystals were cleaved to obtain a clean fresh surface for good ohmic contact. A standard four-point method was employed to eliminate contact resistance with a center electrode pad of 0.5 mm × 0.5 mm^2^ by using sliver paint as an electric contact and gold wire as electric leads. A current of 50 μA was used during all transport measurements.
