# Author Correction: Single Crystal Growth and Spin Polarization Measurements of Diluted Magnetic Semiconductor (BaK)(ZnMn)_2_As_2_

**DOI:** 10.1038/s41598-020-61397-1

**Published:** 2020-03-06

**Authors:** G. Q. Zhao, C. J. Lin, Z. Deng, G. X. Gu, S. Yu, X. C. Wang, Z. Z. Gong, Yasutomo J. Uemura, Y. Q. Li, C. Q. Jin

**Affiliations:** 1 Institute of Physics, Chinese Academy of Sciences; Collaborative Innovation Center of Quantum Matter, Beijing, 100190 China; 2 University of Chinese Academy of Sciences, Beijing, 100190 China; 3 Department of Physics, Columbia University, New York, NY 10027 USA

Correction to: *Scientific Reports* 10.1038/s41598-017-08394-z, published online 03 November 2017

The original version of this Article contained a typographical error in the spelling of the author Yasutomo J. Uemura, which was incorrectly given as Yasutomo J. Uemera This has now been corrected in the PDF and HTML versions of the Article.

